# Rhinitis, sinusitis and ocular disease – 2100. New approach to treat allergic rhinitis with Vitamin E, cod liver oil and Vitamin C with use of nasal steroidal spray

**DOI:** 10.1186/1939-4551-6-S1-P175

**Published:** 2013-04-23

**Authors:** Geetendra Singh Dhanawat

**Affiliations:** 1 Medical Officer, Nabard Regional Office Madhya Pradesh, Bhopal, India

## 

In general practice many of the patients come with a problem of allergic rhinitis and having history of taking medicines like levocetrizine or cetrizine with or without use nasal steroidal spray but the problem persists since several years with little or no improvements. As per my clinical practice I used some new approach with the conventional prescriptions on more than 50 patients who are having problems of allergic rhinitis for more than 5 years. Supplementing with Vitamin E may help relieve some of the symptoms associated with seasonal allergic rhinitis. Allergic rhinitis is an inflammatory condition of the nose, throat, sinuses, and eyes. People with allergic rhinitis may have eye and nose itchiness, nasal stuffiness, episodes of sneezing, and a runny nose. Ear infections and chronic sinusitis may result from long-standing allergic rhinitis, as the passages to the ears and the sinuses become blocked. Vitamin E is a powerful antioxidant, can calm portions of the immune system that are involved in allergic reactions. Cod Liver oil is high in Vitamins A & D which are natural anti-inflammatory to reduce inflammation of the mucus membranes. Vitamin C reduces inflammation and allergic responses. Fish oil may help to decrease the dryness of the lining of the respiratory tract and retain moisture in the the nasal passages. So based on these facts I used a combination of drugs containing Vitamin D, Cod liver oil and High dose of Vitamin B Complex with Monteleukast Sodium & levocetrizine and nasal spray containing Fluticasone Propionate IP with Azelastine Hydrochloride. This therapy gives around >80% relief in symptoms and the frequency of attack significantly reduced. Out of 53 patients 16 have complete relief in three month of therapy than they are switched to multivitamin and multi mineral combinations. Final Conclusion is that in the treatment of allergic rhinitis the role of Vitamins (specially E, C) and Cod liver oil is very significant.

